# The interplay between fast-food outlet exposure, household food insecurity and diet quality in disadvantaged districts

**DOI:** 10.1017/S1368980020004280

**Published:** 2022-01

**Authors:** Laura A van der Velde, Femke MP Zitman, Joreintje D Mackenbach, Mattijs E Numans, Jessica C Kiefte-de Jong

**Affiliations:** 1 Department of Public Health and Primary Care, Leiden University Medical Centre, Albinusdreef 2, 2333 ZA Leiden, The Netherlands; 2 Department of Epidemiology and Biostatistics, Amsterdam University Medical Center, Vrije Universiteit, Amsterdam Public Health Research Institute, Amsterdam, The Netherlands; 3 Department of Epidemiology, Erasmus Medical Center, Rotterdam, The Netherlands

**Keywords:** Fast food, Food security, Food environment, Diet quality, Geographic information systems

## Abstract

**Objective:**

The current study aimed to explore the interplay between food insecurity, fast-food outlet exposure and dietary quality in disadvantaged neighbourhoods.

**Design:**

In this cross-sectional study, main associations between fast-food outlet density and proximity, food insecurity status and dietary quality were assessed using Generalized Estimating Equation analyses. We assessed potential moderation by fast-food outlet exposure in the association between food insecurity status and dietary quality by testing for effect modification between food insecurity status and fast-food outlet density and proximity.

**Setting:**

A deprived urban area in the Netherlands.

**Participants:**

We included 226 adult participants with at least one child below the age of 18 years living at home.

**Results:**

Fast-food outlet exposure was not associated with experiencing food insecurity (fast-food outlet density: *b* = −0·026, 95 % CI = −0·076; 0·024; fast-food outlet proximity: *b* = −0·003, 95 % CI = −0·033; 0·026). Experiencing food insecurity was associated with lower dietary quality (*b* = −0·48 per unit increase, 95 % CI = −0·94; −0·012). This association was moderated by fast-food outlet proximity (*P*_interaction_ = 0·008), and stratified results revealed that the adverse effect of food insecurity on dietary quality was more pronounced for those with the nearest fast-food outlet located closer to the home.

**Conclusions:**

Food insecurity but not fast-food outlet density is associated with dietary quality. However, the association between food insecurity and dietary quality may be modified by the food environment. These findings could inform policymakers to promote a healthier food environment including less fast-food outlets, with particular emphasis on areas with high percentages of food insecure households.

Maintaining a healthy diet is essential for overall health and chronic disease prevention, decreasing the risk of overweight and obesity^(1)^, chronic diseases^(2,3)^ and poor mental health^(4)^. Despite the evident importance of a healthy diet, many people – especially those of lower socio-economic status (SES) groups – find it difficult to meet dietary guidelines^(5)^. Suboptimal food choices result from a combination of personal factors and factors in the physical, social and economic environment^(6)^, such as an unfavourable food environment with high exposure to low-cost, easily accessible fast-foods. Evidence for such an association is inconsistent^(7,8)^, although some evidence suggests that an unfavourable food environment indeed impedes healthy food choices^(9)^.

Previous literature describes five dimensions of the food environment: availability, accessibility, affordability, acceptability and accommodation^(10)^. These first two dimensions (availability and accessibility) reflect geographic distribution^(10)^ and are also important elements of food insecurity, defined as inadequate or insecure access to affordable, healthy foods^(11)^. Narratives of people at risk of food insecurity highlight food outlet availability and accessibility as important factors influencing eating behaviour^(12)^. When budget is limited, accessibility is especially important, as (public) transport can entail additional costs. Another emphasised consideration was food pricing^(12)^, which can be influenced by food outlet density, e.g. due to competitive pricing^(13)^. Also, availability may impact variation in food supply and may therefore influence opportunities for consuming a varied diet.

People experiencing food insecurity may adopt an unfavourable diet with high fast-food intake due to financial constraints, as this kind of diet is generally less expensive than healthier diets^(14)^. Experiencing food insecurity may also indirectly influence food choices through impaired mental health, leading to unfavourable food choices^(12,15)^. These factors help explain why food insecure families tend to have less healthy diets^(16)^. Furthermore, although depending on contextual and individual factors, both food insecurity and fast-food outlets are generally more prevalent in disadvantaged neighbourhoods^(7,17)^. Although mere exposure to fast-food outlets does not necessarily make people eat less healthy^(18)^, it can be speculated that experiencing food insecurity lowers resilience and enhances vulnerability to tempting food cues of low-cost and convenient (fast-)foods^(19)^, and therefore the impact of food outlet exposure on dietary quality could be amplified for those experiencing food insecurity. Ford and Dzewaltowski describe a similar hypothesis after literature review on food environments in the USA, stating that ‘while the quality of the retail food environment affects food choice and eating behaviours among both high and low SES populations, the economic (and perhaps social and cultural) resources available to those of higher SES have a protective effect on eating patterns’^(20, page 225)^. Following this hypothesis, a recent study among a large cohort of adult residents of the United Kingdom showed that those most exposed to fast-food outlets and of lowest SES were most at risk of unhealthy dietary intake and obesity, suggesting a double burden of unfavourable food environments and low SES^(21)^. However, a recent literature review found no clear evidence for a differential impact of food environments on dietary quality across socio-economic groups^(22)^.

All in all, associations between food environments, SES and diet remain complex, and to date only limited research has examined the interplay between fast-food outlet exposure, food insecurity and dietary quality. Therefore, we aimed to explore the interplay between food insecurity, fast-food outlet exposure and dietary quality in disadvantaged neighbourhoods.

## Methods

### Study population and data collection

Participants for our cross-sectional, observational study were recruited between April 2017 and June 2018 in six disadvantaged neighbourhoods in The Hague (see online supplementary material, Supplemental Fig. S1), selected based on predefined criteria of the Dutch Government to identify disadvantaged neighbourhoods^(23)^. Participants who met the inclusion criteria (i.e. living in or near one of the selected neighbourhoods; aged ≥18 years; and having at least one child aged <18 years living at home) were recruited at various public places, such as community centres and (pre)schools. Questionnaires addressing food insecurity status, dietary intake, and sociodemographic variables were available in Dutch, English and Turkish. Participants who provided contact information were contacted to complement missing data from their questionnaire if applicable. A total of *n* 250 participants filled out the questionnaire, of whom 24 were excluded (*n* 8 for having no child <18 years living at home, *n* 16 for having missing postal code data), resulting in a population of analysis of *n* 226 (see online supplementary material, Supplemental Fig. S2). Because the participants’ home postal codes were unevenly distributed over the districts, some districts were merged into larger clusters according to matching neighbourhood characteristics (see online supplementary material, Supplemental Document S1). Participants were placed in one of seven clusters based on their postal code.

### Food insecurity assessment

Household food insecurity status was assessed using the 18-item United States Department of Agriculture Household Food Security Survey Module (USDA-HFSSM)^(24)^, which has a previously confirmed construct validity and reliability^(25)^. Questions addressed household food conditions within the past 12 months. Affirmative responses were summed into an ordinal food insecurity score ranging from 0 to 18. This score was dichotomised into the categories ‘food secure’ (0–2 affirmative responses) and ‘food insecure’ (3–18 affirmative responses)^(26)^. Food insecurity status was analysed continuously (‘food insecurity score’: 0–18) and dichotomously (‘food insecurity status’: food secure/food insecure).

### Dietary quality assessment

Dietary intake was assessed using an adapted version of the Dutch Dietary Quality Screener (Eetscore 1.0), with the previous month as reference period^(27)^. From the dietary intake data, a dietary quality score was constructed assessing adherence to the Dutch dietary guidelines for the following six components: vegetables; fruit; fish; bread; oils and fats; and sweet and savoury snacks. For each component, a minimum score of 0 and a maximum score of 10 could be obtained, with higher scores indicating a better adherence to the dietary guidelines. These component scores were summed, resulting in an overall dietary quality score ranging from 0 to 60. Construction of the dietary quality score is described in more detail elsewhere^(28)^.

### Food outlet exposure assessment

All food outlets in The Hague were extracted from the commercial database Locatus^(29)^, which was recently validated showing good to excellent agreement compared with field audit data^(30)^. Fast-food outlets were classified as shops that sell food which has been prepared in bulk order in advance and which is ordered and paid for at the counter^(31)^. Branch classification codes for fast-food, grillroom/kebab and take-away were used^(18)^. The stores were then geo-located based on their geographical coordinates (see online supplementary material, Supplemental Fig. S1). Food outlet exposure measures were calculated using Geographical Information Systems in Qgis (version 3.8.0-Zanzibar, Free Software Foundation, 1991) using the centre of the 6-digit postal code area (for *n* 35, 6-digit was not available and therefore 4-digit was used). Geographical data for The Hague and the postal code areas were obtained from OpenStreetMap ^(32)^ and the open source Data Platform The Hague^(33)^. We assessed both fast-food outlet proximity (FFP) and fast-food outlet density (FFD) in our study, as these are both important and distinct dimensions of food outlet exposure that may influence eating behaviour of people experiencing food insecurity.

#### Fast-food outlet proximity

Euclidean FFP was calculated as a measure of fast-food accessibility^(34)^. This measure reflects the location of the fast-food outlet and the ease of getting there, expressed in the distance to that location^(8)^. FFP was calculated as the shortest distance from the home postal code to the nearest fast-food outlet, expressed in distance per 10 m to facilitate interpretation of the results.

#### Fast-food outlet density

FFD in a Euclidean buffer of 500 and 1000 m around the home postal code was calculated as a measure of fast-food availability^(34)^, which reflects the adequacy of the variation and amount of food outlets in a certain area^(8)^. The 500 m buffer was chosen as an acceptable walking distance, but analyses with 1000 m buffers were included in sensitivity analyses for comparison, because maximum acceptable walking distance differs per person and per situation.

The number of fast-food outlets correlated strongly with the total number of food outlets in The Hague (Pearson’s rho = 0·919, see online supplementary material, Supplemental Document S2). Therefore, in addition to the *absolute* FFD, we included the *relative* FFD within 500 m as a sensitivity measure to evaluate the effect of the FFD taking into account the total number of food outlets (calculated as: FFD/total number of food outlets).

### Covariates

Socio-demographic characteristics and socio-economic status (SES) proxies were assessed using questionnaires, including age in years; sex (male *v*. female); household size (number of adults and children living in the household); marital status (single *v*. married or cohabiting); migration background (Western *v*. non-Western); educational level (low (≤ISCED 2) *v*. higher (≥ISCED 3)) and gross monthly household income (above *v*. below the Dutch basic needs budget^(35)^). The basic needs budget is calculated taking into account the household size and household composition. To illustrate, the basic needs budget limit is 2235 euro gross monthly income for a two-parent household with two children and 1626 euro for a single-parent household with two children.

### Statistical analysis

Subject characteristics were described as mean and sd or median and interquartile range for continuous variables and percentages for dichotomous variables.

Food insecurity was analysed both continuously (‘food insecurity score’) and dichotomously (‘food insecurity status’). Main associations between FFD and FFP, food insecurity and dietary quality were assessed using Generalized Estimating Equation (GEE) analyses using an exchangeable correlation structure. To assess the association between FFD, FFP and food insecurity, we used GEE analyses with identity link function with food insecurity score as dependent variable and FFD and FFP one by one as independent variables. These analyses were repeated using GEE analyses with logistic link function with food insecurity status as dependent variable. To assess the association between FFD, FFP and dietary quality, we conducted GEE analyses with identity link function, with dietary quality as dependent variable and FFD and FFP one by one as independent variables. To assess the association between food insecurity and dietary quality, we conducted GEE analyses with identity link function, with dietary quality as dependent variable and food insecurity score and food insecurity status one by one as independent variables. All analyses were clustered by district (crude models) and additionally adjusted for age, sex, migration background, household size, marital status, household income and educational level (adjusted model). Potential non-linearity was tested by evaluating a quadratic term.

Further, we tested for a moderating effect of fast-food outlet exposure on the association between food insecurity status and dietary quality by one-by-one adding the interaction terms (1) FFD*food insecurity score; (2) FFP*food insecurity score; (3) FFD*food insecurity status and (4) FFP*food insecurity status to the crude model. If significant interaction was observed, analyses were stratified by the median value for the continuous FFD or FFP. Stratification by the median value was done to obtain two equal-sized subgroups to compare.

Sensitivity analyses were performed conducting the same analyses as described above, but including (1) relative FFD (to explore the effect of taking into account the total number of food outlets); (2) FFD within 1000 m (to explore the effect of a larger exposure radius); (3) only non-foodbank users, as food aid may bias the results and (4) only participants with complete six-digit postal code, as assessments based on four-digit postal code are less accurate.

Missing data were imputed using the multiple imputation procedure in SPSS, using Predictive Mean Matching (*n* 10 imputations). The percentage of missing values ranged between 1·2 and 11·6 % (see online supplementary material, Supplemental Document S3). Results obtained after the multiple imputation procedure are presented.

A two-sided *P*-value of 0·05 was considered statistically significant. Analyses were performed using IBM SPSS statistics version 25.0 (IBM Corp.).

## Results

### Sample characteristics

Overall, 26·5 % of the participants experienced food insecurity (Table 1). The mean (± sd) age was 38·3 (±7·4) years, the majority of participants were women (86·6 %), had a non-Western migration background (84·2 %) and were married or cohabiting (68·2 %). Most participants reported a household income below the basic needs budget (66·6 %) and 58·3 % were higher educated. Only 3·1 % of the participants reported foodbank use. The mean (± sd) dietary quality score was 35·4 (± 7·3) out of 60. Regarding fast-food outlet exposure, the median (interquartile range) FFD within 500 m was 12·0 (6·0; 18·0), meaning that a median number of 12 fast-food outlets were present within a radius of 500 m around the home postal code of the participants. The median (interquartile range) FFP was 139·4 (109·0; 214·3) m, meaning that the median distance from the home postal code of the participants to the closest fast-food outlet was 139·4 m (Table 1). For food insecure participants, the median (interquartile range) FFP was approximately 13 m shorter (131·2 (101·1; 225·7) *v*. 144·6 (108·7; 211·4)), i.e. fast-food outlets were generally 13 m closer to the home postal code of food insecure participants (Table 2).

**Table 1:** Characteristics of included participants (*n* 226)

Characteristics	Mean/median/percentage	sd/IQR
Age (in years)	38·3	7·4
Sex (% women)	86·6 %	
Migration background (% non-Western)	84·2 %	
Household size	4·2	1·3
Marital status (% married or cohabiting)	68·2 %	
Educational level (% higher level, ≥ISCED 3)	58·3 %	
Household income (% below basic needs budget)	66·6 %	
Foodbank users (% yes)	3·1 %	
Total dietary quality score (range 0–60)	35·4	7·3
Food insecurity status (% food insecure)	26·5 %	
Six-digit postal code known (%)	84·5 %	
Total number of places where food is sold within 500 m radius	57·0	26·8; 107·3
Shortest distance from home to fast-food outlet (FFP in metres)	139·4	109·0; 214·3
Number of fast-food outlets within 500 m radius (FFD in 500 m)	12·0	6·0; 18·0
Number of fast-food outlets relative to the total number of food outlets within 500 m radius (relative FFD)	18·2	16·2; 25·0
Number of fast-food outlets within 1000 m radius (FFD in 1000 m)	48·5	25·0; 62·0

sd, standard deviation; IQR, interquartile range; ISCED, International standard classification of education; FFP, fast-food outlet proximity; FFD, fast-food outlet density.

**Table 2:** Median fast-food outlet proximity (FFP) and fast-food outlet density (FFD), for food secure and food insecure participants (*n* 226)

	Food secure		Food insecure	
	Median	IQR	Median	IQR
FFP (shortest distance in m)	144·6	108·7; 211·4	131·2	101·1; 225·7
FFD (in 500 m)	13·0	7·0; 18·0	10·0	6·0; 16·0
Relative FFD (in 500 m)	18·2	16·1; 23·5	19·7	16·4; 26·2
FFD (in 1000 m)	50·0	25·0; 61·3	45·5	22·0; 64·0

IQR, interquartile range.

### Main associations between fast-food outlet exposure, food insecurity and dietary quality

FFP and FFD were not associated with experiencing food insecurity (Table 3). FFD was not associated with dietary quality; however, increasing FFP (i.e. the fast-food outlet being further away from the home postal code) was associated with a slightly higher dietary quality (adjusted model: *b* = 0·12, 95 % CI = 0·025; 0·21). Experiencing food insecurity was significantly associated with lower dietary quality (food insecurity score, adjusted model: *b* = −0·48, 95 % CI = −0·94; −0·012; food insecurity status, adjusted model: *b* = −2·73, 95 % CI = −5·18; −0·29) (Table 3). The multiple imputation procedure had little impact on the observed estimates (see online supplementary material, Supplemental Document S3: Table 4).

**Table 3:** Main associations between fast-food outlet density and proximity, food insecurity and dietary quality (*n* 226)

	Outcome				
	Food insecurity score (continuous)				
	Crude model†			Adjusted model‡	
	*b* §	95 % CI		*b* §	95 % CI
FFD (within 500 m)	−0·023	−0·082; 0·037		−0·026	−0·076; 0·024
FFP (per 10 m)	−0·009	−0·043; 0·025		−0·003	−0·033; 0·026

	Food insecurity status (dichotomous)				
	Crude model†			Adjusted model‡	
	OR‖	95 % CI		OR‖	95 % CI
FFD (within 500 m)	0·98	0·92; 1·04		0·96	0·91; 1·01
FFP (per 10 m)	0·98	0·94; 1·02		0·98	0·95; 1·02

	Dietary quality				
	Crude model†			Adjusted model‡	
	*b* §	95 % CI		*b* §	95 % CI
FFD (within 500 m)	−0·013	−0·17; 0·14		−0·009	−0·16; 0·14
FFP (per 10 m)	0·11	0·014; 0·20*		0·12	0·025; 0·21*
Food insecurity score (continuous)	−0·47	−0·85; −0·093*		−0·48	−0·94; −0·012*
Food insecurity status (dichotomous)	−2·70	−4·47; −0·93*		−2·73	−5·18; −0·29*

FFP, fast-food outlet proximity; FFD, fast-food outlet density.

* *P* < 0·05.

† Crude model: merely including FFD, FFP or food insecurity as determinant, clustered by district (*n* 7).

‡ Adjusted model: crude model additionally adjusted for age, sex, migration background, household size, marital status, household income and educational level.

§ *b* Represents the difference in food insecurity score (higher = more food insecure) or dietary quality (higher = better adherence to dietary guidelines).

‖ OR, odds ratio for being food insecure (being food secure = reference).

### The role of fast-food outlet exposure in the association between food insecurity status and dietary quality

**Fig. 1:**
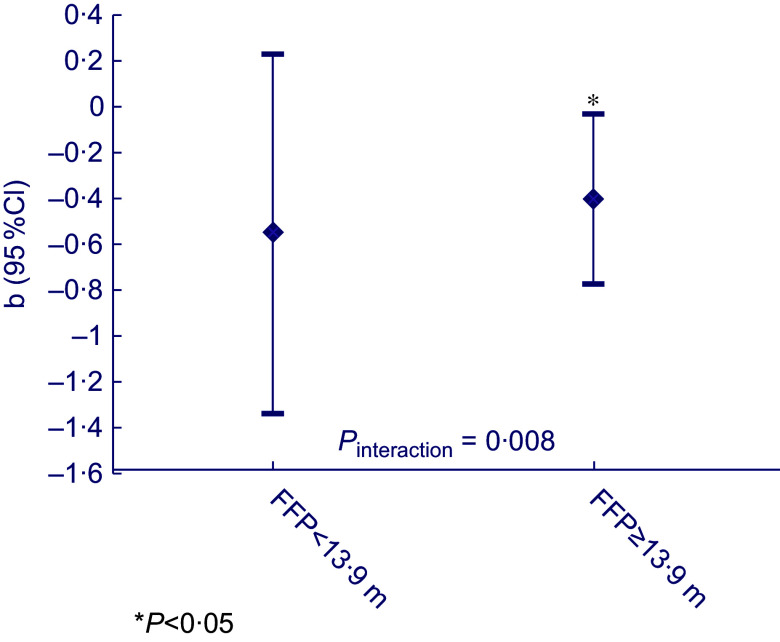
Stratified results for the association between food insecurity score and dietary quality (clustered by district, adjusted for age, sex, migration background, household size, marital status, household income and educational level), split at the median fast-food outlet proximity (FFP) per 10 m: 13·9 m

A significant interaction (*P* = 0·008) was observed for food insecurity score with FFP, whereas no interaction was observed for food insecurity status with FFP (*P*_interaction_ = 0·949) nor for FFD with food insecurity score (*P*_interaction_ = 0·681) or status (*P*_interaction_ = 0·680). Stratification by the population-specific median FFP per 10 m (i.e.13·9 m) showed that for individuals with the nearest fast-food outlet per 10 m being < 13·9 m from the home, a larger effect size was found for the adverse effect of food insecurity on dietary quality (*b* = −0·55, 95 % CI = −1·34; 0·23), whereas for individuals with the nearest fast-food outlet per 10 m being more than 13·9 m from the home, a smaller effect size was observed (*b* = −0·40, 95 % CI = −0·77; −0·031) (Fig. 1).

### Sensitivity analyses

#### Relative fast-food density and fast-food density within 1000 m

Results of the analyses including the *relative* FFD within 500 m or FFD within 1000 m where comparable with the results of the main analyses (see online supplementary material, Supplemental Document S4). Differing from the main analyses, the association between FFD within 1000 m and food insecurity score was significant in the adjusted model, although effect sizes were similar (see online supplementary material, Supplemental Document S4).

#### Non-foodbank users

Sensitivity analyses including only non-foodbank users (*n* 199) showed similar results compared with the main analyses for the associations between FFD and FFP with dietary quality and experiencing food insecurity (see online supplementary material, Supplemental Document S5). For the associations between experiencing food insecurity and dietary quality, effect sizes were smaller but in the same directions. Further, in the analyses including only non-foodbank users, the association between food insecurity and dietary quality was only significant for the crude association between food insecurity status and dietary quality. Stratified results at the median FFP per 10 m were similar to the results of the main analyses for FFP per 10 m ≥ 13·9 m; however, for FFP per 10 m<13·9 m, effect sizes were in the same direction but smaller (see online supplementary material, Supplemental Document S5).

#### Participants that provided complete postal codes

Sensitivity analyses including only the participants that provided their complete 6-digit postal code (*n* 191) showed mostly similar results compared with the main analyses (see online supplementary material, Supplemental Document S6). Differing from the main analyses, the association between FFP and dietary quality was non-significant, slightly smaller effect estimates were observed for the association between food insecurity and dietary quality and the association between experiencing food insecurity and lower dietary quality was only significant for the crude model with the dichotomous food insecurity status (see online supplementary material, Supplemental Document S6).

## Discussion

Our study among families living in an urban multi-ethnic setting in the Netherlands showed that fast-food outlet exposure was not associated with experiencing food insecurity. Increasing FFP was associated with a slightly higher dietary quality. Further, experiencing food insecurity was associated with a lower dietary quality. This association was moderated by FFP, and stratification by the median FFP distance in our sample revealed that the adverse effect of food insecurity on dietary quality was more pronounced for those with the nearest fast-food outlet located closer to the home.

In our study, we did not find an indication that fast-food outlet exposure was related to experiencing food insecurity, suggesting that geographic access to fast-food in this context does not contribute to food insecurity. This could be partly explained by the urban setting in which the study was conducted, where so called ‘food deserts’– areas with poor access to healthy and affordable food – are rare^(36)^. While evidence suggests that food deserts exist in disadvantaged areas in the United States and may there contribute to diet-related health disparities, limited evidence for this phenomenon has been found for other countries including the Netherlands^(36,37)^. Further, our study focussed on access to fast-foods, whereas overall food access is more likely to compromise food security. In addition, food pricing seems to be a more important determinant of food purchase behaviour than food access for low-income and food insecure families^(12,13)^. Therefore, the generally higher prices of healthier diets^(14)^ may explain the association between experiencing food insecurity and a lower dietary quality that was observed in our study. Consistent with our findings, previous literature shows substantial evidence for an association between experiencing food insecurity and lower dietary quality^(16)^, but limited and inconsistent evidence for an association between the food environment and dietary quality^(38)^. Our results indicated that FFD was not related to dietary quality, whereas increasing FFP was associated with a slightly higher dietary quality, indicating that maintaining a healthy diet may be easier when living further away from a fast-food outlet.

In line with our hypothesis, our results showed that the adverse effect of food insecurity on dietary quality was more pronounced among those with the nearest fast-food outlet located closer to the home. Previous literature shows no clear evidence for a differential impact of food environments on dietary quality across socio-economic groups^(22)^. Although food insecurity is more prevalent among lower socio-economic groups, this is not a one-to-one relationship (i.e. not all people with lower incomes experience food insecurity and vice versa). Therefore, it is possible that the impact of food environments on dietary quality indeed is different for those experiencing food insecurity and not for those just belonging to lower socio-economic groups. Narratives of people at risk of experiencing food insecurity, living in the same disadvantaged neighbourhoods as those included in the current study, strengthen our findings as these participants also indicated high fast-food outlet exposure as a barrier for healthy eating^(12)^. It should be noted that we did not observe the same effect modification when we analysed food insecurity status dichotomously instead of assessing food insecurity score. This may be explained by the sample size, but may also suggest a potential plateau effect in which fast-food outlet accessibility interacts with food insecurity and dietary quality. For example, with more severe food insecurity, other (severe) problems such as mental health issues may be more important determinants of dietary quality^(15)^. Future research is warranted to further explore the exact tipping point in food insecurity status were fast-food outlet proximity becomes an important negative influence on dietary quality. The possible implications of our findings are illustrated by the results of a recent longitudinal study, which showed an increase in the availability of food retailers offering convenience and ready-to-eat foods in the Dutch food environment in the past 14 years and higher availability of fast-food outlets in low-SES neighbourhoods^(39)^.

Previous literature suggests that the local retail food environment impacts food choices^(6)^, making the food environment a target for interventions. Geographical Information Systems enable assessment of spatial accessibility to food outlets^(10)^. Dimensions of this geographic accessibility include accessibility of food outlets around the home address^(10)^. The construct of food accessibility is a key element in the official definition of food security defined by the FAO, stating that food security is the ‘physical and economic access to sufficient, safe and nutritious food that meets dietary needs and food preferences for an active and healthy life’^(11)^. However, we used the USDA-HFSS^(24)^, which mostly reflects financial accessibility and is less focused on physical accessibility such as often studied in low-income countries.

Previous studies examining the food environment varied greatly in their methodological choices regarding density/proximity measures, Euclidean/street-network measures, absolute/relative measures, buffer levels and the incorporation of either store prices or people’s store preferences^(34)^. This makes studies on the food environment difficult to compare. The current study contributes to the growing body of literature focused on neighbourhood fast-food environment influences on food insecurity and dietary quality. To our knowledge, this is the first study showing the differential impact of fast-food outlet exposure on dietary quality for those experiencing food insecurity.

Strengths of the current study include the use of both proximity and density measures for quantifying fast-food outlet exposure and the performance of sensitivity analyses using the relative density and density within a larger radius. This allowed comprehensive analyses and better understanding of the actual associations with fast-food outlet exposure. Further, our study was strengthened by methodological correction using multiple imputation to account for potential bias associated with missing data^(40)^.

Limitations of the current study include the relatively small sample size. Our power calculation was initially based on a sample of 250 participants, whereas in the current study some participants were excluded resulting in a slightly smaller sample size of 226 participants. Therefore, null findings need to be interpreted with caution.

Because of the cross-sectional design of the current study, it was not possible to infer causal or directional relationships. In addition, a potential effect of residential self-selection cannot be ruled out. Residential self-selection indicates that the selection of a neighbourhood to live in may be related to the neighbourhood exposure (such as the food environment) and the health outcome of interest (such as diet quality)^(41)^, which may lead to biased results^(42)^. For example, if participants have a preference for fast-food restaurants, they may have selected the neighbourhoods they lived in for its fast-food outlet presence, while this preference may also negatively impact diet quality. On the other hand, participants may have selected the disadvantaged neighbourhoods they lived in because of financial constraints, while fast-food restaurants are also generally more prevalent in these neighbourhoods^(7)^. The most common method to account for residential self-selection is model adjustment, as was performed in our study^(42)^. Although we have adjusted our analyses for various factors including household income, it should be noted that other factors influencing neighbourhood choice may not have been accounted for, such as personal preference for a certain food environment.

Another potential drawback is that we focused exclusively on the food outlet exposure surrounding the participants’ home and did not take into account other relevant food outlet exposure such as those surrounding the worksite, while clearly these places could add to the food outlet exposure^(43)^. In addition, we assessed fast-food outlet exposure, but we had no information on if and where fast-food was actually purchased or consumed. Therefore, future studies that include a more comprehensive assessment of all relevant fast-food outlet exposure and taking into account actual food purchase and consumption behaviour are warranted to confirm our results. It should further be noted that we based our dietary quality score on Dutch dietary guidelines, which may be less suitable for non-Dutch ethnic groups. In addition, the dietary quality score did not reflect fast-food consumption specifically, but rather reflected overall dietary quality. Also, we used the USDA-HFSSM to assess food insecurity status, which is regarded as the golden standard for Western countries^(44)^ but is not yet validated for the Dutch population.

## Conclusions

In conclusion, our study indicated that fast-food outlet exposure was not associated with experiencing food insecurity. Experiencing food insecurity was associated with a lower dietary quality, and the adverse effect of food insecurity on dietary quality was more pronounced for those with the nearest fast-food outlet located closer to the home. Future research is warranted to further explore the role of fast-food outlet exposure in the association between food insecurity and dietary quality and the exact tipping point in food insecurity status where fast-food outlet proximity becomes an important negative influence on dietary quality, especially in light of the increasing availability of fast-food outlets in low-SES neighbourhoods. If our findings are confirmed by future studies, these results could inform policymakers to promote a healthier food environment including less fast-food outlets, with particular emphasis on areas with high percentages of food insecure households, as this might be a promising strategy for improving dietary quality among those households and thereby reduce health disparities.

## Supplementary material

For supplementary material accompanying this paper visit https://doi.org/10.1017/S1368980020004280. click here to view supplementary material
