# Supplementary material for: The interplay between fast-food outlet exposure, household food insecurity and diet quality in disadvantaged districts
Source: Public Health Nutr. 2020 Oct 27;25(1):105–13. doi: 10.1017/S1368980020004280 (PMC8825968; doi:10.1017/S1368980020004280)
Supplement: Supplementary file 1 [file S1368980020004280sup001.pdf]

# Supplementary material

For the manuscript:

**‘The interplay between fast-food outlet exposure, household food insecurity and diet quality in disadvantaged districts’**

## Table of Contents

**Figure S1**. Locations (green-yellow dots) were fast-food is sold in The Hague, according to the Locatus database ........ 2

**Figure S2.** Flow chart presenting recruitment, inclusion and exclusion of participants ........ 3

**Document S1.** Clustering of districts ........ 4

**Document S2.** Fast-food outlets and the total number of food outlet locations in The Hague ........ 6

**Document S3.** Details of the multiple imputation procedure for missing values ........ 7

**Document S4.** Main associations between the relative fast-food density within 500 meter and the absolute fast-food density within 1000 meter, food insecurity and dietary quality ........ 11

**Document S5 .** Sensitivity analyses including only non-foodbank users........ 13

**Document S6.** Sensitivity analyses including only participants that provided their full 6-digit postal code........ 16

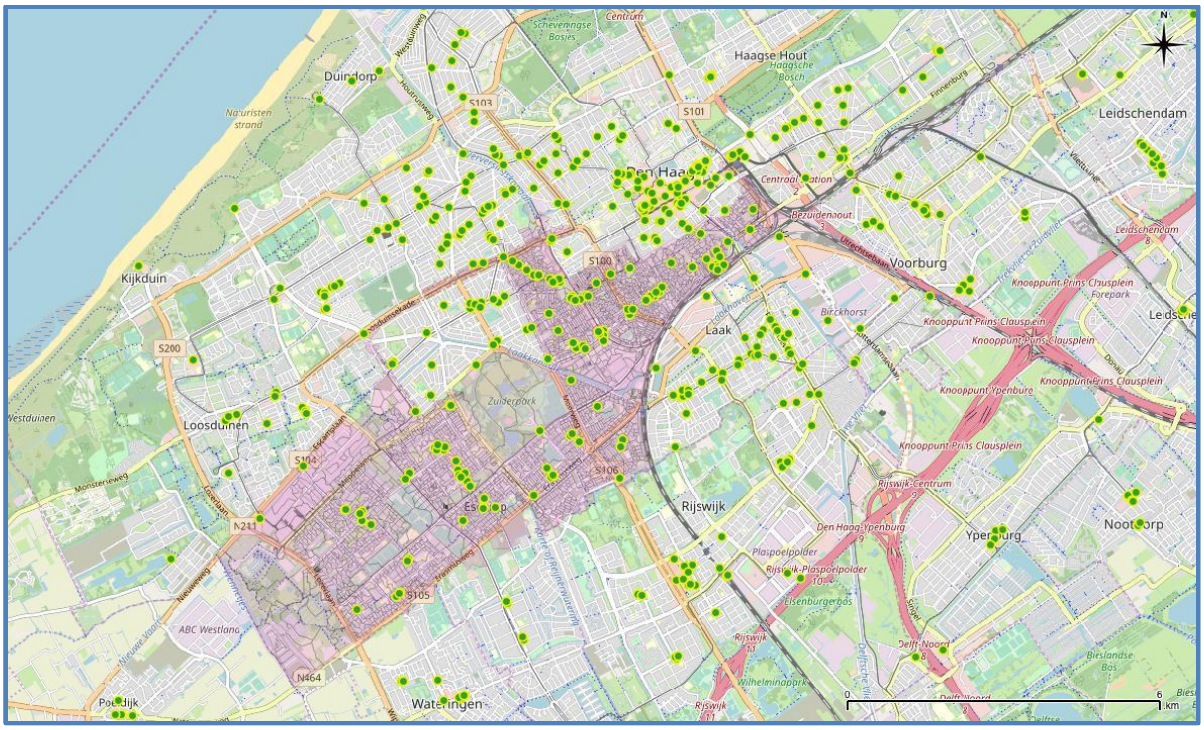


**Figure S1**. Locations (green-yellow dots) were fast-food is sold in The Hague, according to the Locatus database (selected for the following branches: fast-food, grillroom/kebab and delivery/take out). The six disadvantaged neighbourhoods are highlighted in purple.

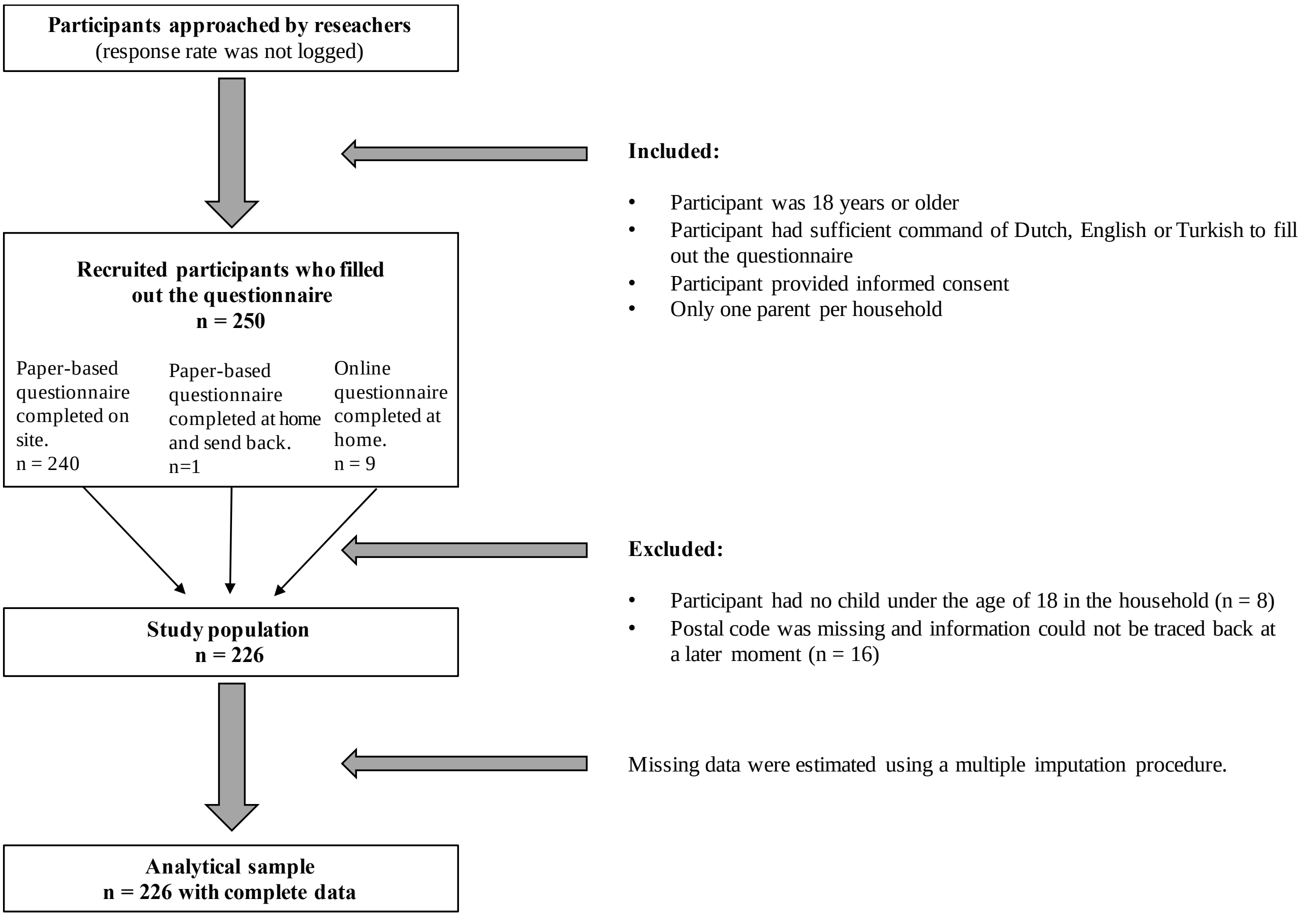


**Figure S2.** Flow chart presenting recruitment, inclusion and exclusion of participants

## **Document S1.** Clustering of districts

The 226 participants included in the current study that provided their postal code could be assigned to one of 16 districts in the Dutch city The Hague. Districts are presented outlined in purple in **Document S1: Figure 1**. However, the participants' households were unevenly distributed over the districts (**Document S1: Table 1**), which could bias the results. Districts were therefore merged into 7 larger clusters, with at least 20 participants in each of the clusters. The cluster number for each district is also presented in **Document S1: Figure 1**. When districts were merged, this was done based on neighborhood characteristics (Foundation living in The Hague 2019 (in Dutch: "Stichting wonen in Den Haag 2019"), date cited: 7-8-2019, available from: https://wonenindenhaag.nl), as summarized in **Document S1: Table 1**.

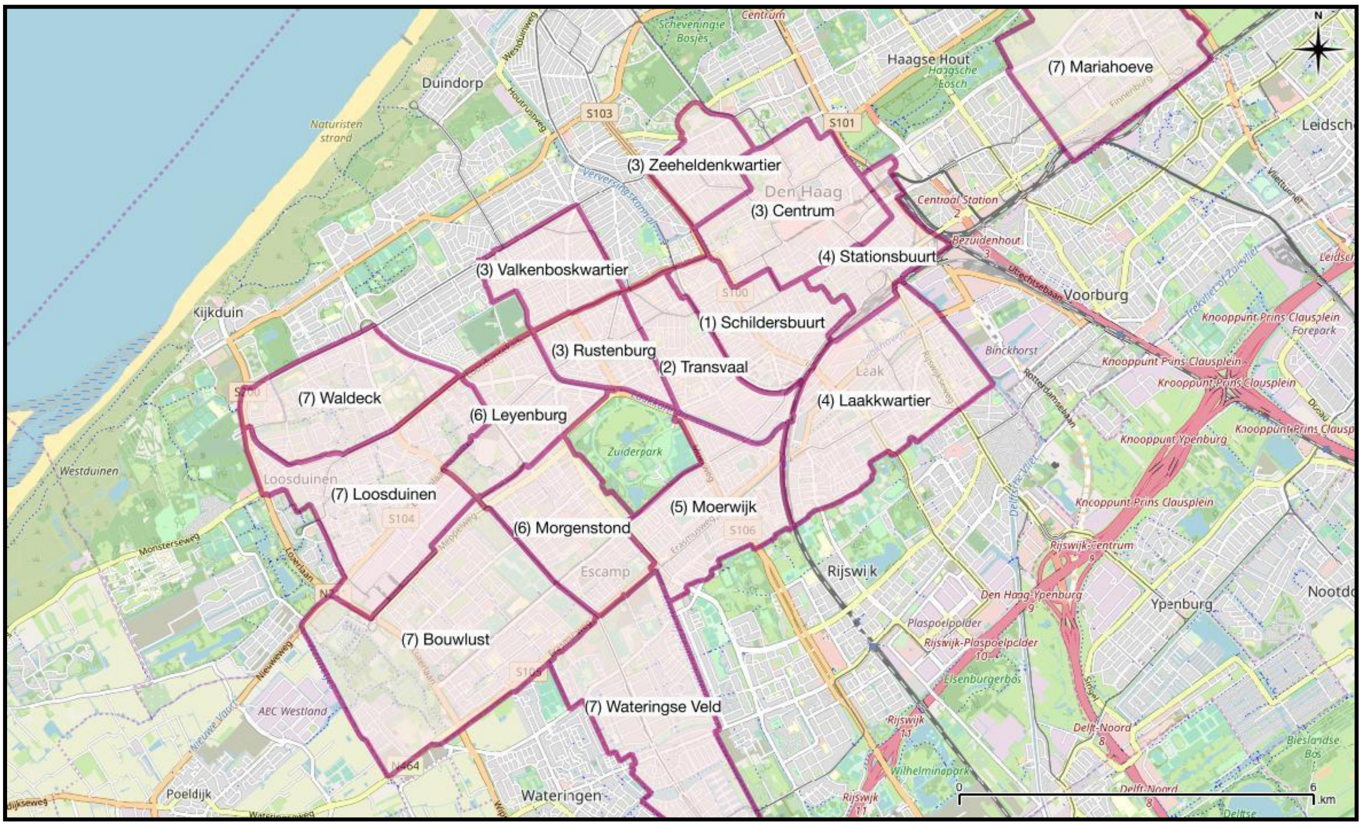


**Document S1: Figure 1.** *The districts with the number of the cluster they belong to (1 to 7) between brackets.*

**Document S1: Table 1**. Characteristics of the seven clusters (n=226).

| Cluster number | Total nr of participants | Included districts | Nr of participants per district | Merging criteria |
|---|---|---|---|---|
| 1 | 56 | Schildersbuurt | 56 | NA * |
| 2 | 41 | Transvaal | 41 | NA * |
| 3 | 30 | Centrum | 18 | High number of shops |
| | | Zeeheldenkwartier | 1 | High number of shops |
| | | Rustenburg | 5 | High number of shops |
| | | Valkenboskwartier | 6 | High number of shops |
| 4 | 23 | Laakkwartier | 8 | Near train stations |
| | | Stationsbuurt | 15 | Near train stations |
| 5 | 29 | Moerwijk | 29 | NA * |
| 6 | 21 | Morgenstond | 17 | Adjacent to Zuiderpark |
| | | Leyenburg | 4 | Adjacent to Zuiderpark |
| 7 | 26 | Wateringseveld | 2 | Green and spacious neighborhoods |
| | | Bouwlust | 21 | Green and spacious neighborhoods |
| | | Loosduinen | 1 | Green and spacious neighborhoods |
| | | Waldeck | 1 | Green and spacious neighborhoods |
| | | Mariahoeve | 1 | Green and spacious neighborhoods |

** Not applicable with only 1 district in this cluster.*

## **Document S2.** Fast-food outlets and the total number of food outlet locations in The Hague

The number of fast-food outlets was highly correlated with the total number of food outlets in The Hague (Pearson's rho = 0.919), as shown in **Document S2: Figure 1**. All food outlets in The Hague were extracted from the Locatus database (Locatus (2019). *Retail Facts*. Available from: https://locatus.com). Analyses were performed using Qgis (version 3.8.0-Zanzibar, Free Software Foundation, 1991, Boston USA).

The total number of food outlets within 500m from the center of each 6-digit postal code area (n=14726) included the following branches for food outlets:

| | | | |
|---|---|---|---|
| • Hotel-restaurant | • Pies / flans | • Restaurant | • Fruit and vegetables |
| • Lunchroom | • Coffee / tea | • Fast-food | • Toko |
| • Café-restaurant | • Cheese | • Grillroom/kebab | • Deli |
| • Fish | • Nuts | • Cafe | • Night shop |
| • Butchery | • Reform | • Baker | • Poulterer |
| • Take away / delivery | • Sweets | • Wine shop | • Supermarket |
| • Coffee shop | • Food public transport | • Ice cream shop | • Hospital shop |
| • Mini supermarket | • Catering public transport | • Chocolate | • Shisha lounge |
| • Liquor store | | | |

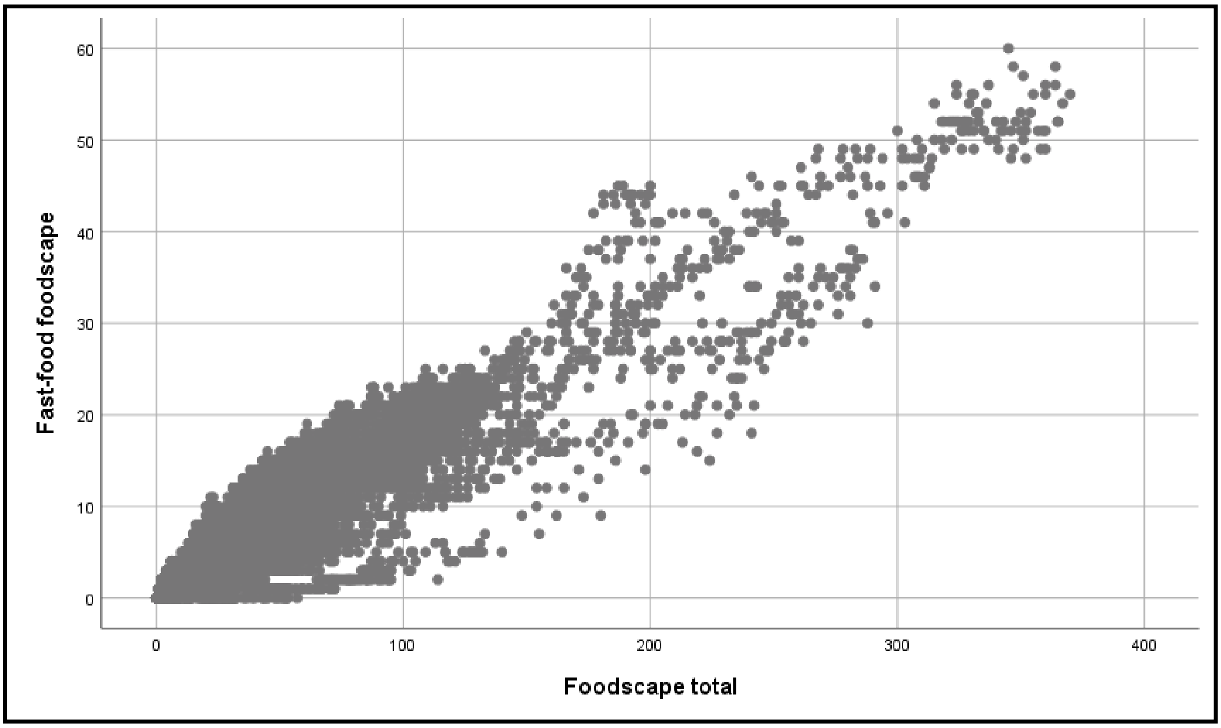


**Document S1: Figure 1**. *Graphic representation of the relation between the number of fast-food outlets and the total number of food outlets within a 500m radius of the center of all 6-digit postal code areas in and around the Dutch city The Hague*

**Document S3.** Details of the multiple imputation procedure for missing values

Missing data were analyzed and addressed using the multiple imputation technique in SPSS. Selected variables for imputation are summarized in **Document S3: Table 1**. A separate variance *t*-test was used for variables with more than 5% missing data: the mean dietary quality score for the present and missing selection was significantly different for the variables household size and household income, but not for age. This suggests that data is missing at random, which is a rationale for imputation and offers opportunities for prediction of missing data. Household income was the variable with the highest number of missing values (28 out of 242, see **Document S3: Table 1**).

**Document S3: Table 1.** Missing data (t-test for variables with more than 5% missing values)

| | Numbers | | Separate variance *t*-test for Dietary quality score | | |
|---|---|---|---|---|---|
| **Variables** | **Missing** | **Present** | **Missing** | **Present** | ***p-value*** |
| Age (years) | 13 | 229 | 34.4 | 35.4 | 0.642 |
| Sex (male/ female) | 3 | 239 | | | |
| Migration background (Western/ non-Western) | 4 | 238 | | | |
| Household size | 13 | 229 | 29.8 | 35.7 | 0.001 |
| Marital status (single/ married or cohabiting) | 8 | 234 | | | |
| Educational level (≤ ISCED-2/ ≥ ISCED-3) | 8 | 234 | | | |
| Household income (below/ above basic needs level) | 28 | 214 | 31.4 | 35.9 | 0.001 |

Imputation was performed including the 16 participants who did not provide their postal code, i.e. 242 participants were taken into account. To increase prediction power, 70 variables from the original dataset (derived from the complete questionnaire) were used as predictors. These variables are summarized in **Document S3: Table 1** (n= 7) and **Document S3: Table 2** (n= 63).

The missing data were estimated using the Predictive Mean Matching method in SPSS with ten sets of imputations with a maximum of 50 iterations (seed was set at 950 on beforehand). The pooled results of these imputations were used in the analyses described in the main manuscript. This document shows the results for the original (non-imputed) data for the 226 participants who could be geo-located in one of the districts in The Hague and were included in the current study. **Document S3: Table 3** shows the descriptive analyses of the variables in the original and the imputed data: changes due to imputation were relatively small, with an uppermost increase of 8% for household income. **Document S3: Table 4** show results from the same analyses as presented in the main manuscript (**Table 3**), in the original and imputed data.  Similar effect sizes were observed for these analyses in original and imputed data. (**Document S3: Table 4**).

**Document S3: Table 2.** Variables used as predictors in the imputations (excluding the predictors that were also imputed, those are presented in **Document S3: Table 1**).

| **Variables used as predictors** | | |
|---|---|---|
| • Length | • Weight | • Pregnancy status |
| • Number of adults in the house | • Number of children in the house | • Marital status (5 categories) |
| • Country where you were born | • Country where father is born | • Country where mother is born |
| • Religion | • Currently employed | • Employed in the past |
| • Currently smoking | • Smoked in the past | • How much do you smoke a day |
| • What do you smoke | • Do you buy food at the supermarket | • Do you buy food at the Turkish supermarket |
| • Do you buy food at the market | • Do you buy food at the deli | • Do you make use of the foodbank |
| • High blood pressure | • Blood pressure medication | • High cholesterol |
| • Cholesterol medication | • Cardiac treatment | • Open heart surgery |
| • Heart attack | • Asthma | • COPD |
| • Lung medication | • Diabetes | • Type of diabetes |
| • Do you use insulin for your diabetes | • Do you use tablets for your diabetes | • Anemic |
| • Do health issues hinder you with the shopping | • Number of days a week physical activity | • Minutes a day physical activity |
| • Food security questionnaire - question 1 | • Food security questionnaire - question 2 | • Food security questionnaire - question 3 |
| • Food security questionnaire - question 4 | • Food security questionnaire - question 5 | • Food security questionnaire - question 6 |
| • Food security questionnaire - question 7 | • Food security questionnaire - question 8 | • Food security questionnaire - question 9 |
| • Food security questionnaire - question 10 | • Food security questionnaire - question 11 | • Food security questionnaire - question 12 |
| • Food security questionnaire - question 13 | • Food security questionnaire - question 14 | • Food security questionnaire - question 15 |
| • Food security questionnaire - question 16 | • Dietary quality score (6 components) | • Location of sampling |
| • Number of fast-food outlets in 500m radius | • Number of fast-food outlets in 1000m radius | • Distance to nearest fast-food outlet |
| • Postal code | • District | • Cluster |

**Document S3: Table 3.** Characteristics of included participants, in original and imputed data

| | Original data | | Imputed data | |
|---|---|---|---|---|
| | | Number of missings | | Number of missings |
| Age (in years) | 38.3 (±7.4) | 5 | 38.3 (7.4) | 0 |
| Sex (% women) | 86.3% | 2 | 86.6% | 0 |
| Migration background (% non-Western) | 84.1% | 1 | 84.2% | 0 |
| Household size | 4.2 (±1.3) | 10 | 4.2 (1.3) | 0 |
| Marital status (% married or cohabiting) | 66.4% | 6 | 68.2% | 0 |
| Educational level (% lower level) | 40.1% | 8 | 41.7% | 0 |
| Household income (% below basic needs budget) | 61.1% | 23 | 66.6% | 0 |
| Total score dietary quality (range 0-60) | 35.4 (±7.3) | 0 | 35.4 (7.3) | 0 |
| Food security (% food insecure) | 26.5% | 0 | 26.5% | 0 |

*Numbers are means (±SD) or percentages.*

**Document S3: Table 4**. Main associations between fast-food outlet density and proximity, food insecurity and dietary quality in original and imputed data (n=226)

| | **Original data** | | | | **Imputed data** | | | |
|---|---|---|---|---|---|---|---|---|
| | **Outcome** | | | | **Outcome** | | | |
| | **Food insecurity score (continuous)** | | | | **Food insecurity score (continuous)** | | | |
| | Crude model | | Adjusted model | | Crude model | | Adjusted model | |
| | b | 95% CI | b | 95% CI | b | 95% CI | b | 95% CI |
| **FFD** (within 500 m) | -0.023 | -0.082; 0.037 | -0.026 | -0.076; 0.024 | -0.023 | -0.082; 0.037 | -0.026 | -0.076; 0.024 |
| **FFP** (per 10 m) | -0.009 | -0.043; 0.025 | 0.00 | -0.033; 0.033 | -0.009 | -0.043; 0.025 | -0.003 | -0.033; 0.026 |
| | **Food insecurity status (dichotomous)** | | | | **Food insecurity status (dichotomous)** | | | |
| | Crude model | | Adjusted model | | Crude model | | Adjusted model | |
| | OR | 95% CI | OR | 95% CI | OR | 95% CI | OR | 95% CI |
| **FFD** (within 500 m) | 0.98 | 0.92; 1.04 | 0.95 | 0.91; 1.00* | 0.98 | 0.92; 1.04 | 0.96 | 0.91; 1.01 |
| **FFP** (per 10 m) | 0.98 | 0.94; 1.02 | 0.99 | 0.95; 1.02 | 0.98 | 0.94; 1.02 | 0.98 | 0.95; 1.02 |
| | **Dietary quality** | | | | **Dietary quality** | | | |
| | Crude model | | Adjusted model | | Crude model | | Adjusted model | |
| | b | 95% CI | b | 95% CI | b | 95% CI | b | 95% CI |
| **FFD** (within 500 m) | -0.013 | -0.167; 0.141 | -0.079 | -0.21; 0.054 | -0.009 | -0.16; 0.14 | -0.022 | -0.17; 0.13 |
| **FFP** (per 10 m) | 0.11 | 0.014; 0.201* | 0.10 | 0.021; 0.19* | 0.12 | 0.025; 0.21* | 0.11 | 0.017; 0.20* |
| **Food insecurity score (continuous)** | -0.47 | -0.85; -0.093* | -0.38 | -0.80; -0.052 | -0.48 | -0.94; -0.012* | -0.49 | -0.95; -0.028* |
| **Food insecurity status (dichotomous)** | -2.70 | -4.47; -0.93* | -2.50 | -4.55; -0.46* | -2.73 | -5.18; -0.29* | -2.89 | -5.33; -0.45* |

** p < 0.05*

*95% CI = 95% confidence interval*

*OR= odds ratio for being food insecure (being food secure=reference)*

*b represents the difference in food insecurity score (higher= more food insecure) or dietary quality (higher=better adherence to dietary guidelines)*

*Crude model: Merely including FFD, FFP or food insecurity as determinant, clustered by district (n=7)*

*Adjusted model: Crude model additionally adjusted for age, sex, migration background, household size, marital status, household income, and educational level*

## **Document S4.** Main associations between the relative fast-food density within 500 meter and the absolute fast-food density within 1000 meter, food insecurity and dietary quality

In addition to the analyses with absolute FFD within a 500m radius, we also performed the analyses with the relative FFD and FFD within a 1000m radius. For the association with food insecurity score (continuous), similar to the results of the main analyses including the absolute FFD within 500m, the *relative* FFD within 500m was not significantly associated with experiencing food insecurity, although effect sizes were larger and in the opposite direction (*relative* FFD within 500m, Adjusted model: b=0.031, 95%CI=-0.004; 0.066; *absolute* FFD within 500m, Adjusted model: b= -0.026, 95%CI=-0.076; 0.024). For the FFD within 1000m effect sizes were similar compared to the main analyses with absolute FFD within 500m, but significant in the adjusted model (Adjusted model: b=-0.017, 95%CI=-0.032; -0.001) (**Document S4: Table 1; Main manuscript: Table 3**).

For the association with food insecurity status (dichotomous), similar to the results of the main analyses including the absolute FFD within 500m, the relative FFD and FFD within 1000m were not significantly associated with experiencing food insecurity, with odds ratio's  around 1 (**Document S4: Table 1**).

For the association with dietary quality, similar to the results of the main analyses including the absolute FFD within 500m, the relative FFD and FFD within 1000m were not significantly associated with dietary quality (**Document S4: Table 1**).

Similar to the results of the analyses including the absolute FFD within 500m, no significant interaction between food insecurity status and relative FFD within 500m (continuous score: p=0.841, dichotomous status: p=561) or FFD within 1000m (continuous score: p=0.807; dichotomous status: p=760) was found (data not shown).

**Document S4: Table 1.** Main associations between the relative fast-food density within 500 meter and the absolute fast-food density within 1000 meter, food insecurity and dietary quality (n=226)

| | Outcome | | | |
|---|---|---|---|---|
| | **Food insecurity score (continuous)** | | | |
| | Crude model | | Adjusted model | |
| | b | 95% CI | b | 95% CI |
| **Relative FFD** (within 500m) | 0.040 | -0.005; 0.086 | 0.031 | -0.004; 0.066 |
| **Absolute FFD** (within 1000m) | -0.012 | -0.031; 0.006 | -0.017 | -0.032; -0.001* |
| | **Food insecurity status (dichotomous)** | | | |
| | Crude model | | Adjusted model | |
| | OR | 95% CI | OR | 95% CI |
| **Relative FFD** (within 500m) | 1.02 | 0.98; 1.05 | 1.01 | 0.98; 1.05 |
| **Absolute FFD** (within 1000m) | 1.00 | 0.98; 1.01 | 0.99 | 0.97; 1.00 |
| | **Dietary quality** | | | |
| | Crude model | | Adjusted model | |
| | b | 95% CI | b | 95% CI |
| **Relative FFD** (within 500m) | -0.072 | -0.22; 0.080 | -0.076 | -0.20; 0.048 |
| **Absolute FFD** (within 1000m) | 0.013 | -0.025; 0.051 | 0.016 | -0.014; 0.046 |

** $p < 0.05$*
*95% CI = 95% confidence interval*
*OR= odds ratio for being food insecure (being food secure=reference)*
*b represents the difference in food insecurity score (higher= more food insecure) or dietary quality (higher=better adherence to dietary guidelines)*
*Crude model: Merely including Relative FFD (within 500m) or Absolute FFD (within 1000m) as determinant, clustered by district (n=7)*
*Adjusted model: Crude model additionally adjusted for age, sex, migration background, household size, marital status, household income, and educational level*

## **Document S5 .** Sensitivity analyses including only non-foodbank users

Only 7 participants answered the question regarding foodbank services use affirmative. However, for an additional 20 participants their answer was missing. We performed sensitivity analyses excluding all participants that either answered to be foodbank users or did not answer the question about foodbank use. **Document S5: Table 1** presents the main associations between fast-food outlet density and proximity, food insecurity and dietary quality for non-foodbank users. For the associations between FFD and FFP with dietary quality and experiencing food insecurity, effect sizes closely resembled the results of the analyses were all participants were included (**Main manuscript: Table 3**).

For the associations between experiencing food insecurity and dietary quality, effect sizes were smaller but in the same directions compared to the main analyses including all participants (**Document S5: Table 1**; **Main manuscript: Table 3**). Further, the results including all participants showed a significant association between experiencing food insecurity and lower dietary quality in all models, whereas in the analyses including only non-foodbank users this association was only significant for the crude association between food insecurity status (dichotomous) and dietary quality (b=-2.40, 95%CI=-4.79; -0.009) (**Document S5: Table 1**; **Main manuscript: Table 3**).

Similar to the results presented in the main manuscript including all participants, a significant interaction (p=0.001) was observed for food insecurity score (continuous) with FFP, whereas no significant interaction was observed for food insecurity status (dichotomous) with FFP nor for food insecurity (both continuous and dichotomous) with FFD (**Document S5: Table 2**).

Stratified results at the median FFP per 10m were similar to the results of the main analyses including all participants for FFP per 10 m≥13.9m, however, for FFP per 10m<13.9m effect sizes were in the same direction but smaller compared to the results of the main analyses including all participants (**Document S5: Table 2; Main manuscript: Figure 1**).

**Document S5: Table 1**. Main associations between fast-food outlet density and proximity, food insecurity and dietary quality, analyses including only non-foodbank users (n=199)

| | Outcome | | | |
|---|---|---|---|---|
| | **Food insecurity score (continuous)** | | | |
| | Crude model | | Adjusted model | |
| | b | 95% CI | b | 95% CI |
| **FFD** (within 500 m) | -0.024 | -0.075; 0.027 | -0.024 | -0.067; 0.020 |
| **FFP** (per 10 m) | -0.013 | -0.018; 0.022 | -0.005 | -0.035; 0.026 |
| | **Food insecurity status (dichotomous)** | | | |
| | Crude model | | Adjusted model | |
| | OR | 95% CI | OR | 95% CI |
| **FFD** (within 500 m) | 0.98 | 0.93; 1.04 | 0.98 | 0.93; 1.02 |
| **FFP** (per 10 m) | 0.97 | 0.94; 1.01 | 0.98 | 0.94; 1.02 |
| | **Dietary quality** | | | |
| | Crude model | | Adjusted model | |
| | b | 95% CI | b | 95% CI |
| **FFD** (within 500 m) | -0.008 | -0.20; 0.19 | -0.007 | -0.19; 0.18 |
| **FFP** (per 10 m) | 0.13 | 0.040; 0.21* | 0.14 | 0.047; 0.23* |
| **Food insecurity score (continuous)** | -0.41 | -0.85; 0.018 | -0.38 | -0.88; 0.11 |
| **Food insecurity status (dichotomous)** | -2.40 | -4.79; -0.009* | -2.32 | -5.24; 0.60 |

** p < 0.05;*

*95% CI = 95% confidence interval*

*OR= odds ratio for being food insecure (being food secure=reference)*

*b represents the difference in food insecurity score (higher= more food insecure) or dietary quality (higher=better adherence to dietary guidelines)*

*Crude model: Merely including FFD, FFP or food insecurity as determinant, clustered by district (n=7)*

*Adjusted model: Crude model additionally adjusted for age, sex, migration background, household size, marital status, household income, and educational level*

**Document S5: Table 2.** Stratified results for the association between food insecurity and dietary quality, split at the median fast-food outlet proximity (FFP) per 10m: 13.9m, analyses including only non-foodbank users (n=199)

| | **FFP per 10 m <13.9m** | | **FFP per 10 m ≥13.9m** | | |
|---|---|---|---|---|---|
| **Food insecurity score (continuous)** | | | | | |
| | b | 95%CI | b | 95%CI | |
| **Crude model** | -0.50 | -1.06; 0.068 | -0.36 | -0.79; 0.066 | p-interaction[1]=0.001 |
| **Adjusted model** | -0.33 | -1.16; 0.49 | -0.38 | -0.81; -0.056 | |
| **Food insecurity status (dichotomous)** | | | | | |
| | b | 95%CI | b | 95%CI | |
| **Crude model** | -1.75 | -5.66; 2.16 | -3.13 | -5.11; -1.34* | p-interaction[2]=0.592 |
| **Adjusted model** | -1.01 | -5.55; 3.54 | -3.52 | -6.04; -1.00* | |

**p<0.05*

*[1] Interaction term= FFP per 10 m * **continuous** food insecurity score*

*[2] Interaction term= FFP per 10 m* **dichotomous** food insecurity status*

*b represents the difference in dietary quality score with increasing food insecurity (i.e., being more food insecure)*

*Crude model: Merely including food insecurity status as determinant, clustered by district (n=7)*

*Adjusted model: Crude model additionally adjusted for fast-food outlet density (FFD) within 500m, age, sex, migration background, household size, marital status, household income, and educational level*

## **Document S6.** Sensitivity analyses including only participants that provided their full 6-digit postal code

Not all participants provided their full 6-digit postal code (comprising 4 numbers and 2 letters): for n=35 participants the two letters were missing. A 4-digit postal code can be used to assign the home of a participant to a neighborhood, but this is far less accurate compared to the 6-digit postal code. A sensitivity analysis including only the participants that provided their full 6-digits postal code (85.5% of the study population) was performed to examine whether the results in the main analyses (**Main manuscript: Table 3**) were influenced by a decreased accuracy due to the n=35 incomplete (4-digit) postal codes.

**Document S6: Table 1** presents the main associations between fast-food outlet density and proximity, food insecurity and dietary quality for participants that provided their full 6-digit postal code. For the associations between FFD and FFP with dietary quality and experiencing food insecurity, effect sizes closely resembled the results of the main analyses were all participants were included, although the association between FFP and dietary quality was non-significant when only participants that provided their full 6-digit postal code were included (**Document S6: Table 1; Main manuscript: Table 3**).

For the associations between experiencing food insecurity and dietary quality, effect sizes were slightly less strong but in the same directions compared to the main analyses including all participants (**Document S6: Table 1**; **Main manuscript: Table 4**). Further, the results including all participants showed a significant association between experiencing food insecurity and lower dietary quality in all models, whereas in the analyses including only participants that provided their full 6-digit postal code this association was only significant for the crude and adjusted associations between food insecurity status (dichotomous) and dietary quality (Adjusted model: b-2.45, 95%CI=-4.44; -0.47) (**Document S6: Table 1**; **Main manuscript: Table 3**).

Similar to the results presented in the main manuscript including all participants, a significant interaction (p=0.019) was observed for food insecurity score (continuous) with FFP, whereas no significant interaction was observed for food insecurity status (dichotomous) with FFP nor for food insecurity (both continuous and dichotomous) with FFD (**Document S6: Table 2**).

Stratified results at the median FFP per 10m were similar to the results of the main analyses including all participants (**Document S6: Table 2; Main manuscript: Figure 1**).

**Document S6: Table 1**. Main associations between fast-food outlet density and proximity, food insecurity and dietary quality, analyses including only participants that provided their full 6-digit postal code (n=191)

| | **Outcome** | | | |
|---|---|---|---|---|
| | **Food insecurity score (continuous)** | | | |
| | Crude model | | Adjusted model | |
| | b | 95% CI | b | 95% CI |
| **FFD** (within 500 m) | -0.026 | -0.099; 0.047 | -0.029 | -0.086; 0.028 |
| **FFP** (per 10 m) | -0.01 | -0.045; 0.025 | -0.001 | -0.033; 0.032 |
| | **Food insecurity status (dichotomous)** | | | |
| | Crude model | | Adjusted model | |
| | OR | 95% CI | OR | 95% CI |
| **FFD** (within 500 m) | 0.98 | 0.91; 1.04 | 0.97 | 0.91; 1.03 |
| **FFP** (per 10 m) | 0.98 | 0.94; 1.02 | 0.99 | 0.95; 1.02 |
| | **Dietary quality** | | | |
| | Crude model | | Adjusted model | |
| | b | 95% CI | b | 95% CI |
| **FFD** (within 500 m) | 0.008 | -0.121; 0.137 | 0.006 | -0.12; 0.13 |
| **FFP** (per 10 m) | 0.078 | -0.02; 0.176 | 0.087 | -0.006; 0.18 |
| **Food insecurity score** (continuous) | -0.42 | -0.84; 0.012 | -0.44 | -0.96; 0.086 |
| **Food insecurity status** (dichotomous) | -2.45 | -4.44; -0.47* | -2.56 | -5.21; 0.087 |

** $p < 0.05$;*

*95% CI = 95% confidence interval*

*OR= odds ratio for being food insecure (being food secure=reference)*

*b represents the difference in food insecurity score (higher= more food insecure) or dietary quality (higher=better adherence to dietary guidelines)*

*Crude model: Merely including FFD, FFP or food insecurity as determinant, clustered by district (n=7)*

*Adjusted model: Crude model additionally adjusted for age, sex, migration background, household size, marital status, household income, and educational level*

**Document S6: Table 2**. Stratified results for the association between food insecurity and dietary quality, split at the median fast-food outlet proximity (FFP) per 10m: 13.9m, analyses including only participants that provided their full 6-digit postal code (n=191)

| | FFP per 10 m <13.9m | | FFP per 10 m ≥13.9m | | |
|---|---|---|---|---|---|
| **Food insecurity score (continuous)** | | | | | |
| | b | 95%CI | b | 95%CI | |
| **Crude model** | -0.60 | -1.18; -0.012* | -0.30 | -0.72; 0.12 | p-interaction[1]=0.019 |
| **Adjusted model** | -0.56 | -1.49; 0.36 | -0.36 | -0.78; -0.065 | |
| **Food insecurity status (dichotomous)** | | | | | |
| | b | 95%CI | b | 95%CI | |
| **Crude model** | -2.08 | -5.61; 1.46 | -2.95 | -4.93; -0.98* | p-interaction[2]=0.911 |
| **Adjusted model** | -1.43 | -6.15; 3.29 | -3.53 | -5.82; -1.25* | |

**p<0.05*

*[1] Interaction term= FFP per 10 m * **continuous** food insecurity score*

*[2] Interaction term= FFP per 10 m* **dichotomous** food insecurity status*

*b represents the difference in dietary quality score with increasing food insecurity (i.e., being more food insecure)*

*Crude model: Merely including food insecurity status as determinant, clustered by district (n=7)*

*Adjusted model: Crude model additionally adjusted for fast-food outlet density (FFD) within 500m, age, sex, migration background, household size, marital status, household income, and educational level*
